# Supplementary material for: Characterization of Chromatin Accessibility in Fetal Bovine Chondrocytes
Source: Animals (Basel). 2023 Jun 5;13(11):1875. doi: 10.3390/ani13111875 (PMC10251841; doi:10.3390/ani13111875)

Figure S1. The size of fragments generated by transposase. The horizontal axis shows the fragment length (bp); The vertical axis shows the normalized read density (×10-3).

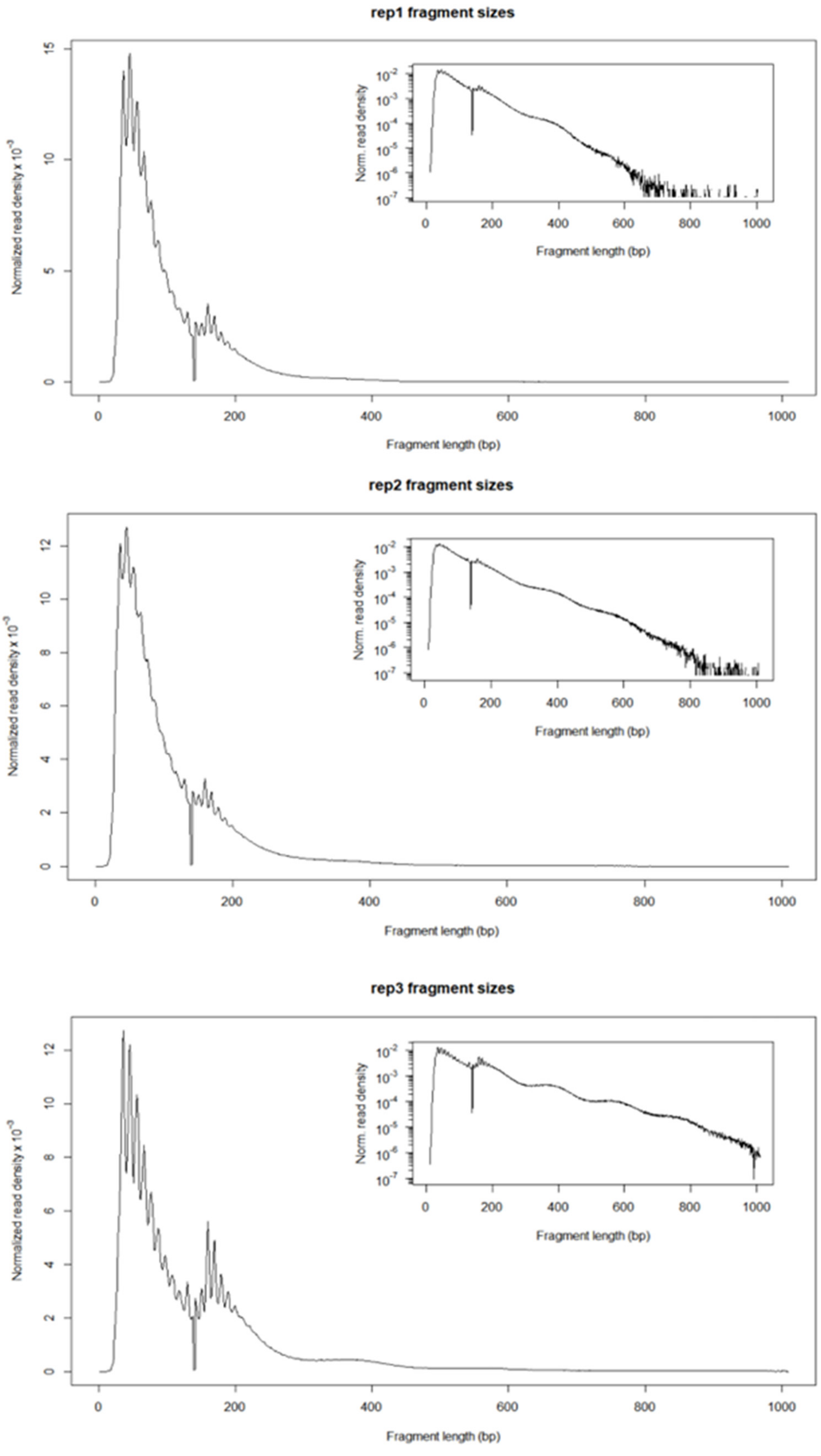

Supplement: Supplementary file 1 [file animals-13-01875-s001.zip › animals-2372865-supplementary.pdf]
